# Mitochondrial Mutations in Subjects with Psychiatric Disorders

**DOI:** 10.1371/journal.pone.0127280

**Published:** 2015-05-26

**Authors:** Adolfo Sequeira, Brandi Rollins, Christophe Magnan, Mannis van Oven, Pierre Baldi, Richard M. Myers, Jack D. Barchas, Alan F. Schatzberg, Stanley J. Watson, Huda Akil, William E. Bunney, Marquis P. Vawter

**Affiliations:** 1 Functional Genomics Laboratory, Department of Psychiatry & Human Behavior, University of California Irvine, Irvine, California, United States of America; 2 School of Information and Computer Sciences (ICS), Institute for Genomics and Bioinformatics (IGB), University of California Irvine, Irvine, California, United States of America; 3 Department of Forensic Molecular Biology, Erasmus MC, University Medical Center Rotterdam, Rotterdam, the Netherlands; 4 HudsonAlpha Institute for Biotechnology, Huntsville, Alabama, United States of America; 5 Department of Psychiatry, Weill Cornell Medical College, New York, New York, United States of America; 6 Department of Psychiatry & Behavioral Sciences, Stanford University, Palo Alto, California, United States of America; 7 Molecular and Behavioral Neurosciences Institute, University of Michigan, Ann Arbor, Michigan, United States of America; 8 Department of Psychiatry & Human Behavior, University of California Irvine, Irvine, California, United States of America; RIKEN Brain Science Institution, JAPAN

## Abstract

A considerable body of evidence supports the role of mitochondrial dysfunction in psychiatric disorders and mitochondrial DNA (mtDNA) mutations are known to alter brain energy metabolism, neurotransmission, and cause neurodegenerative disorders. Genetic studies focusing on common nuclear genome variants associated with these disorders have produced genome wide significant results but those studies have not directly studied mtDNA variants. The purpose of this study is to investigate, using next generation sequencing, the involvement of mtDNA variation in bipolar disorder, schizophrenia, major depressive disorder, and methamphetamine use. MtDNA extracted from multiple brain regions and blood were sequenced (121 mtDNA samples with an average of 8,800x coverage) and compared to an electronic database containing 26,850 mtDNA genomes. We confirmed novel and rare variants, and confirmed next generation sequencing error hotspots by traditional sequencing and genotyping methods. We observed a significant increase of non-synonymous mutations found in individuals with schizophrenia. Novel and rare non-synonymous mutations were found in psychiatric cases in mtDNA genes: ND6, ATP6, CYTB, and ND2. We also observed mtDNA heteroplasmy in brain at a locus previously associated with schizophrenia (T16519C). Large differences in heteroplasmy levels across brain regions within subjects suggest that somatic mutations accumulate differentially in brain regions. Finally, multiplasmy, a heteroplasmic measure of repeat length, was observed in brain from selective cases at a higher frequency than controls. These results offer support for increased rates of mtDNA substitutions in schizophrenia shown in our prior results. The variable levels of heteroplasmic/multiplasmic somatic mutations that occur in brain may be indicators of genetic instability in mtDNA.

## Introduction

The mitochondrial hypothesis of psychiatric disorders derives from evidence of energy metabolism alterations, high prevalence of affective disorders in patients with mitochondrial disorders, and from increased maternal heritability [1]. Cross-sectional risk studies have revealed a significantly higher risk for schizophrenia in relatives who shared mitochondrial DNA (mtDNA) with a schizophrenia patient [2]. However, studies concentrating on major mtDNA haplogroups have failed to reveal clear differences between these major haplogroups in terms of risk to develop psychiatric disorders [3–7]. Recent studies have also suggested that variants in mtDNA can contribute to the risk to develop major depressive disorder (MDD), bipolar disorder (BD) and schizophrenia (SZ) [8–13]. Additionally, some patients with mitochondrial disorders caused by known mtDNA mutations often present psychiatric symptoms [14–16], suggesting a major role of mtDNA mutations in the predisposition to psychiatric disorders. Incidentally, in a large population analysis, common mtDNA variants have been shown to also increase the risk of many seemingly unrelated diseases, some affecting the brain such as ischaemic stroke, multiple sclerosis and Parkinson’s disease [13].

The mitochondrial DNA (mtDNA) is a 16.6 kb circular molecule maternally transmitted and located inside the mitochondrion. The main role of mitochondria is to produce energy through oxidative phosphorylation (OXPHOS). The mtDNA genome encodes 13 OXPHOS proteins, 22 tRNAs, and 12S and 16S rRNA genes. Because each cell contains between 100 to 1000 mitochondria, and each mitochondrion contains a variable number of mtDNA molecules [17, 18], mtDNA mutations can be homoplasmic (present in all copies of the mtDNA genome) or heteroplasmic, with mutations only present in a fraction of the mtDNA molecules. In the past, cloning and Sanger sequencing have been used to investigate heteroplasmy levels, but recent next-generation sequencing (NGS) advancements now allow the study of mtDNA variation with sufficient coverage to uncover heteroplasmy [9, 19, 20].

Genetic predisposition for psychiatric disorders has been extensively studied but few candidate gene variants have been validated across cohorts. However, most of these studies have focused on nuclear genes instead of mtDNA variants. The mitochondrial genome is particularly sensitive to oxidative stress and tends to accumulate somatic mutations with age, particularly in high energy demanding regions such as the brain. Chronic methamphetamine (METH) use is also associated with increased oxidative stress and mitochondrial dysfunction [21]. We therefore included a group of METH users to investigate the chronic effects of this drug on somatic mutations in METH susceptible regions of the brain.

Prior studies, using NGS, have shown an increase in somatic homoplasmic and heteroplasmic mtDNA mutations in cancer [22], cardiomyopathy [23], and aging [24, 25]. We recently used NGS to investigate the involvement of mtDNA somatic homoplasmic mutations in a small sample of brains from patients with psychiatric disorders [9].

In this study, we investigated the involvement of homoplasmic, multiplasmic and heteroplasmic variation in mtDNA from 69 subjects, using NGS on 11 brain regions and blood samples from patients with psychiatric conditions and normal controls. Our working hypothesis was that a greater number of mtDNA mutations would occur in cases compared to controls. We also hypothesized that somatic mutations can appear in some brain regions and accumulate to a deleterious level and play a role in the pathophysiology of psychiatric disorders. Brain tissue is a unique resource to investigate the occurrence of heteroplasmic mutations not necessarily present in peripheral tissues such as blood.

## Results

We analyzed 121 complete mtDNA sequences from 69 subjects, including samples from several brain regions and from blood for three subjects (**S1 and S2 Tables**). All 121 mtDNA sequences passed stringent quality control and were deposited at NCBI (http://www.ncbi.nlm.nih.gov/) accession numbers KC257284-KC257404. Despite differences in overall coverage, reflecting differences between the two platforms efficiency and our multiplexing, we did not observe major differences in the variants reported by the two Illumina platforms (GAII (cohort 1) and HiSeq (cohort 2)). GAII produced an average coverage for the variants of 3,766 (min = 100, max = 15,620) and the HiSeq platform an average of 9,775 (min = 1,114, max = 107,710), with a combined overall average coverage of 8,850.

### Mitochondrial DNA variation

Of the 3,670 mtDNA variants across the 121 samples, the majority were homoplasmic (3177) and 493 were heteroplasmic. The majority of heteroplasmic variants were C>T or A>G transitions, consistent with the expected transition to transversion rate [26].

Consensus mtDNA sequences of the 121 samples were used to build a phylogenetic tree (**S1 Fig**) following established PhyloTree topology and haplogroup nomenclature^24^. Subjects were distributed across diverse haplogroups with no clear clustering of diagnosis, suggesting that there is no specific increase in the predisposition to psychiatric disorders in mitochondria haplogroups (**S1 Fig**). Given the perfect agreement of consensus sequences across brain regions and blood, we decided to focus on the DLPFC data to compare subjects based on diagnosis. We observed a total of 1748 sequence variants in the DLPFC from 63 unique subjects, but many of them were haplogroup specific and reflected divergence from the mitochondrial revised Cambridge Reference Sequence (rCRS; GenBank accession number NC_012920)[27]/. The rCRS, often used as the reference, is a useful tool to compare mitochondrial genomes but does not represent the most common haplotype or an ancestral haplotype, it is simply one haplotype. One subject with schizophrenia, for instance, was identical to the rCRS, while a normal control of African American ancestry carried a large number of divergent loci compared to the rCRS. We therefore excluded the major haplogroup defining variants to explore the specific involvement of mitochondrial variation in psychiatric disorders. A total of 1,175 variants in the DLPFC were further investigated, 984 were homoplasmic (**S3 Table**) and mainly located in the hypervariable region of the mtDNA, and 191 were heteroplasmic (see section regarding heteroplasmic variation).

### Homoplasmic variants

Of the 984 homoplasmic variants, 141 were located within genes and were non-synonymous, therefore potentially functional (**S4 Table**). Comparison of variants only observed in cases or only observed in controls revealed 49 non-synonymous variants (37 loci) only observed among 43 cases (**Table 1**) versus 12 non-synonymous variants only present in the 20 controls. Of these 37 loci, a total of 8 were predicted using Polyphen as being possibly/probably damaging mutations. There were 80 shared variants between cases and controls. The ratio of the number of non-synonymous mutations to genomes sequenced revealed a significantly higher (p = 0.024) number of mutations in SZ (1.57) versus controls (0.55) (**Table 2**). We next tested whether the distribution of the total number of non-synonymous mutations in cases was different compared to controls and found a non-significant trend for five or more non-synonymous mutations in cases compared to controls (p = 0.068, **S5 Table**). We found six homoplasmic non-synonymous mutations that have not been previously reported in MITOMAP, mtDB, or PhyloTree (**Table 3**). Two of these mutations were only found in an online ancestry database (http://www.ianlogan.co.uk/mtdna.htm) but were not present in the PhyloTree database containing 16,810 mtDNA sequences as of September 2012 [28]. Of these six non-synonymous novel and rare variants, four were located in ND6, ATP6, CYTB and ND2, and were only observed in cases while several mutations located in ATP6 (Tyr212His, Asn39Thr and Met140Thr) but present both in controls and cases, which were predicted to be damaging by Polyphen.

**Table 1:** Non-synonymous homoplasmic substitutions at 37 loci were found only present in cases but not in control DLPFC samples.

Position	Gene	BD	MDD	SZ	Total	Reference	Observed	AA Change	Prediction	Haplogroup	MitoMap (N)	SwissProt
3509*	ND1		1		1	T	C	Ile68Thr	benign	H61	3	P03886
3796	ND1		1	1	2	A	G	Thr164Ala	benign	H1b1, H1b1	157	P03886
3992	ND1			2	2	C	T	Thr229Met	benign	H4a1a1a, H2a2a1	212	P03886
4024	ND1			2	2	A	G	Thr240Ala	benign	H4a1a1a, H2a2a1	166	P03886
4025	ND1			1	1	C	T	Thr240Met	benign	H3h	254	P03886
4561	ND2		1		1	T	C	Val31Ala	benign	K2a10	221	P03891
4732	ND2			1	1	A	G	Asn88Ser	benign	U5b2a1a1b	198	P03891
**4824**	**ND2**	**1**		**1**	**2**	**A**	**G**	**Thr119Ala**	**possibly damaging**	**A2d, A2p**	**746**	**P03891**
4924*	ND2		1		1	G	C	Ser152Thr	benign	U5a1d2b	7	P03891
5073^**#**^	ND2			1	1	A	G	Ile202Val	benign	K1b2b	1	P03891
5277	ND2			1	1	T	C	Phe270Leu	benign	A2p	75	P03891
5913	COX1			1	1	G	A	Asp4Asn	benign	K1b2b	191	P00395
6366	COX1	1			1	G	A	Val155Ile	benign	A2d	98	P00395
6480	COX1		1		1	G	A	Val193Ile	benign	I2d	70	P00395
8108	COX2	1			1	A	G	Ile175Val	benign	A2d	45	P00403
8463*	**ATP8**	**1**			**1**	**A**	**G**	**Tyr33Cys**	**probably damaging**	**X2c1b**	**8**	**P03928**
**8519**	**ATP8**		**1**		**1**	**G**	**A**	**Glu52Lys**	**possibly damaging**	**I4a**	**63**	**P03928**
8794	ATP6	1		1	2	C	T	His90Tyr	benign	A2d, A2p	726	P00846
**8843**	**ATP6**	**1**			**1**	**T**	**C**	**Ile106Thr**	**possibly damaging**	**T2b4**	**119**	**P00846**
**9055**	**ATP6**		**2**	**1**	**3**	**G**	**A**	**Ala177Thr**	**possibly damaging**	**K2a10, K1b2b, K1a3a**	**1401**	**P00846**
**9160** ^**#**^	**ATP6**	**1**	** **	** **	**1**	**T**	**C**	**Tyr212His**	**probably damaging**	**A2d**	**1**	**P00846**
9210	COX3			1	1	A	G	Thr2Ala	benign	H1ag	33	P00414
11016	ND4	1			1	G	A	Ser86Asn	benign	H48	184	P03905
11204	ND4	1			1	T	C	Phe149Leu	benign	H1	100	P03905
12346	ND5			1	1	C	T	His4Tyr	unknown	U2e1c	168	P03915
12397	ND5	1			1	A	G	Thr21Ala	unknown	X2a2	134	P03915
12811	ND5			1	1	T	C	Tyr159His	benign	H3h	214	P03915
**13117**	**ND5**		**1**		**1**	**A**	**G**	**Ile261Val**	**possibly damaging**	**K1a3a**	**47**	**P03915**
13637	ND5			1	1	A	G	Gln434Arg	benign	U5b2a1a1b	243	P03915
13708	ND5	1		1	2	G	A	Ala458Thr	benign	J1c2o, U5b2a1a1b	1912	P03915
14110	ND5		1		1	T	C	Phe592Leu	benign	H1	247	P03915
14280^**#**^	ND6			1	1	A	C	Ser132Ala	benign	U2e1c	2	P03923
14502	ND6	1			1	T	C	Ile58Val	benign	X2a2	111	P03923
14582	ND6			2	2	A	G	Val31Ala	benign	H4a1a1a, H2a2a1	164	P03923
14798	CYTB	1	2	1	4	T	C	Phe18Leu	benign	J1c2o, K2a10, K1b2b, K1a3a	2114	P00156
**14982** ^**#**^	**CYTB**		**1**		**1**	**T**	**C**	**Ile79Thr**	**possibly damaging**	**V16**	**2**	**P00156**
15431	CYTB	1			1	G	A	Ala229Thr	benign	I3b	326	P00156

The observed haplogroups are randomly distributed. The effect of the amino acid substitutions was determined using Polyphen, damaging mutations are shown in bold.

^*^ Frequency corresponds to less than 0.1% of 26,850 human mtDNA sequences with size greater than 15.4 kbp collected from GenBank on 25 June 2014.

^#^ Mutations were found in Mitomap (accessed June 2014) as a result of our NCBI data deposit.

**Table 2:** The number of non-synonymous mutations shown in S4 Table, was extracted when present only in controls and not cases with psychiatric disorders, and is shown in the row for control subjects.

	Subjects	Mutations	Mutations/subject	z-score
Controls	20	11	0.55	-0.990
BD	14	14	1.00	-0.056
MDD	15	13	0.87	-0.333
SZ	14	22	1.57	1.380

The number of non-synonymous mutations found only in cases (and not controls) is shown on each row for each disorder. The ratio of the number of mutations / subject was calculated for each group, and a z-score for the difference of the observed for each group compared to the entire mean was calculated. There was a significant increase in non-synonymous mutations (p = 0.024, two tailed z-score test) in persons with SZ compared to controls.

**Table 3:** Novel-rare NS mutations observed in 65 DLPFC brain samples and confirmed by Sanger sequencing, the D-loop mutation is non-coding.

Gene	Mutation	Coverage	Amino Acid Change	Age	Gender	Axis I	Haplogroup	Status
ATP6	T8945C* ^1^	2820	Met140Thr-damaging	55	M	C	H1	Rare
ATP6	A8642C	2572	Asn39Thr-damaging	54	M	C	I1	Novel
ATP6	T9160C	28247	Tyr212His-damaging	50	M	BD	A2d	Novel
CYTB	T14982C*	12465	Ile79Thr-moderate	41	F	MDD	V16	Rare
ND6	A14280C	1113	Ser132Ala-benign	36	M	SZ	U2e1c	Novel
ND2	A5073G	20128	Ile202Val-benign	40	F	SZ	K1b2b	Novel
D-loop	T16178G	22475	-	35	M	SZ	A2p	Novel

The effect of the amino acid substitutions was determined using Polyphen.

*Once in submitted online mtDNA sequencing data.

^1^ T8945C was previously reported by our group [9].

Recently, whole genome sequencing studies have shown increased mutation rates in schizophrenia but only examined nuclear genes [29, 30]. In the current study, we have focused on the occurrence of nonsynonymous variants in mtDNA as another distinct possible genetic predisposition to schizophrenia.

Several mutations in ribosomal RNA (rRNA) were also observed only in cases not in controls (**S6 Table** in gray). The 16S rRNA had 10 variants only observed in cases and the 12S rRNA had 6 variants that were only observed in cases. The z-score difference between the number of unique rRNA variants for controls and schizophrenia was not significant (p = 0.15). Two of these rRNA variants were rare mutations present only in an MDD subject (C1601T) and in a BD subject (T1861C). After querying multiple online databases these mutations were found via Ian Logan’s website in accessions HM238202 (Philippines; haplogroup B4a1a) and JN872374 (Italy; haplogroup U1a3). The C1601T mutation occurs at the 3’ terminus of the 12S mtDNA rRNA and does not appear to pose functional effects, while the T1861C mutation occurs at a non-complementary bridge, that increases complementarities in that region.

### Heteroplasmic variation in mtDNA

We observed a total of 114 heteroplasmic variants in 69 unique mtDNA genomes. Heteroplasmic mutations were defined as any variant for which the major allele was <95% of the total of the observed alleles and a minimum coverage of 200 reads. As expected, the heteroplasmic variants were mainly clustered within the hypervariable region (**S7 Table**). No clear over-representation of heteroplasmic mutations was observed in cases versus controls (except for the multiplasmy described below). Four heteroplasmic variants with high heteroplasmy and two multiplasmic variants were successfully validated by Sanger sequencing, confirming the reliability of next generation sequencing and of our quality control criteria for the investigation of heteroplasmic mtDNA variation (**Table 4**).

**Table 4:** Heteroplasmy and multiplasmy in mtDNA.

	Subject	Haplogroup	Region	Diagnosis	Gender	Age	Position	Reference	Alleles	Heteroplasmy	Gene	Status
**Heteroplasmy**	C-58	L1c3b2	All*	C	F	58	11696^#^	G	G/A	24.8–28.3%	ND4	Known
	C-58	L1c3b2	All*	C	F	58	16086	T	T/C	67.4–93.7%	D-loop	Known
	M-18	N1a1a1a2	DLPFC	MDD	M	35	16086	T	T/C	85.5%	D-loop	Known
	M-18	N1a1a1a2	DLPFC	MDD	M	35	16261	C	C/T	70.9%	D-loop	Known
	S-111	H56	DLPFC	SZ	M	47	16519	T	T/C	18.9%	D-loop	Novel
	D-84	H69	DLPFC	Meth	F	34	16519	T	T/C	90.0%	D-loop	Novel
**Multiplasmy**	C-25	H5d	All*	C	M	64	514	(CA)_5_	(CA)_4,5_		D-loop	Known
	C-58	L1c3b2	All*	C	F	58	514	(CA)_5_	(CA)_4,5_		D-loop	Known
	C-83	U5b1e1	DLPFC	C	M	44	514	(CA)_5_	(CA)_4,5_		D-loop	Known
	B-71	X2a2	DLPFC	BD	F	70	514	(CA)_5_	(CA)_4,5_		D-loop	Known
	**B-76**	**I3b**	**DLPFC**	**BD**	**M**	**39**	**514**	**(CA)**_**5**_	**(CA)**_**5,6,7**_		**D-loop**	**Known**
	B-78	A2d	DLPFC	BD	M	50	514	(CA)_5_	(CA)_4,5_		D-loop	Known
	B-79	H1	DLPFC	BD	M	43	514	(CA)_5_	(CA)_4,5_		D-loop	Known
	D-85	A2c	SN	Meth	M	35	514	(CA)_5_	(CA)_4,5_		D-loop	Known
	D-86	H2a2b1a	SN	Meth	F	39	514	(CA)_5_	(CA)_5,6_		D-loop	Known
	M-96	I4a	DLPFC	MDD	M	61	514	(CA)_5_	(CA)_4,5_		D-loop	Known
	M-97	H1b1	DLPFC	MDD	F	63	514	(CA)_5_	(CA)_4,5_		D-loop	Known
	M-98	HV	DLPFC	MDD	F	41	514	(CA)_5_	(CA)_4,5_		D-loop	Known
	S-110	U5b2a1a1b	DLPFC	SZ	F	41	514	(CA)_5_	(CA)_5,6_		D-loop	Known
	S-106	H4a1a1a	DLPFC	SZ	M	59	514	(CA)_5_	(CA)_4,5_		D-loop	Known
	S-107	H2a2a1	DLPFC	SZ	M	45	514	(CA)_5_	(CA)_4,5_		D-loop	Known
	S-112	A2p	DLPFC	SZ	M	35	514	(CA)_5_	(CA)_4,5_		D-loop	Known
	**S-114**	**K1b2b**	**DLPFC**	**SZ**	**F**	**40**	**514**	**(CA)**_**5**_	**(CA)**_**5,6,7**_		**D-loop**	**Known**
	C-83E	U5b1e1	DLPFC	C	M	44	D16189	(C)_10_	(C)_9,10,11_		D-loop	Known
	C-90E	B2g	DLPFC	C	F	32	D16189	(C)_10_	(C)_9,10,11_		D-loop	Known
	C-89E	H1ap1	DLPFC	C	M	68	D16189	(C)_10_	(C)_9,10,11_		D-loop	Known
	B-77E	H1b	DLPFC	BD	M	50	D16189	(C)_10_	(C)_9,10,11_		D-loop	Known
	B-71E	X2a2	DLPFC	BD	F	70	D16189	(C)_10_	(C)_9,10,11_		D-loop	Known
	M-97E	H1b1	DLPFC	MDD	F	63	D16189	(C)_10_	(C)_9,10,11_		D-loop	Known

**Fig 1:**
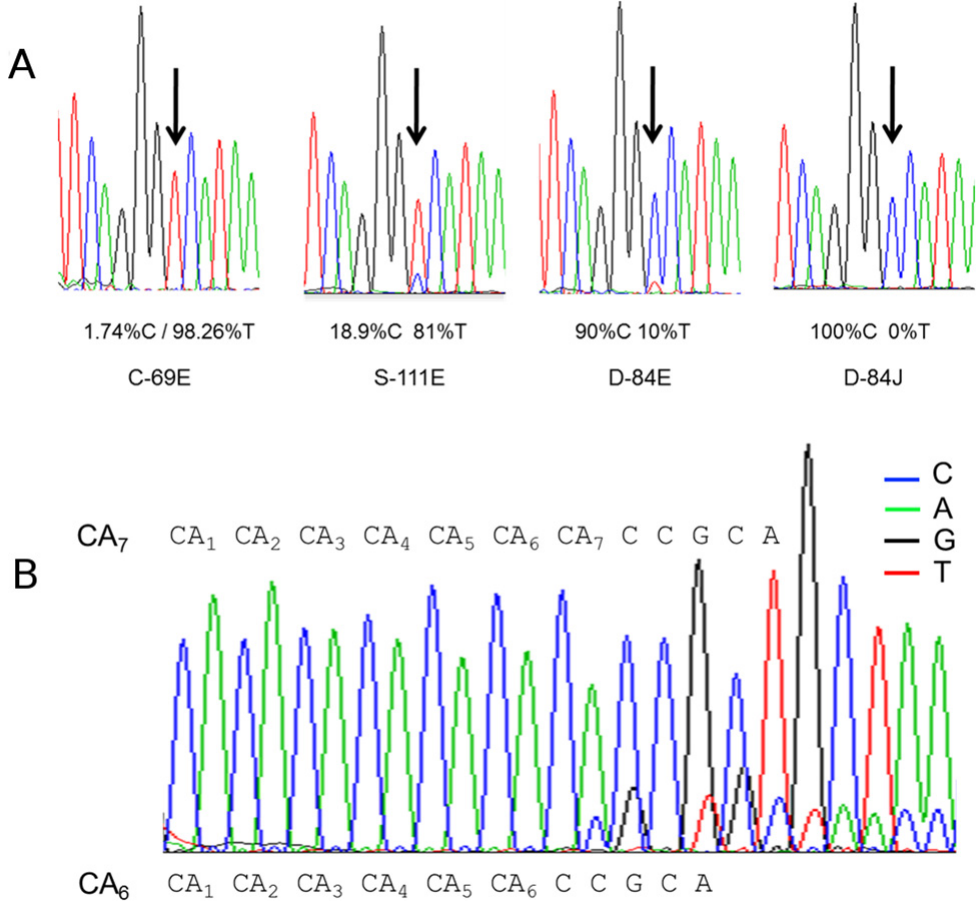
Electropherograms confirming heteroplasmy. **A)** Heteroplasmy at the 16519 locus confirmed by Sanger sequencing and showing clear reversals of allele calls and heteroplasmy concordant with NGS calculated results. The electropherograms for D-84 are from the same subject but from two different brain regions, D-84E corresponds to DLPFC with 10% T reads and D-84J corresponds to substantia nigra SN with 0% T reads. **B)** Multiplasmy confirmed by Sanger sequencing. Electropherogram showing a psychiatric subject with 5, 6 and 7 (CA)n dinucleotide repeats at position 514 in the mtDNA displacement Loop. The actual number of repeats was determined from the individual reads from the sequencing data, 5 and 6 repeats are over-imposed in the electropherogram, consistent with the NGS results.

The heteroplasmy was calculated from NGS reads and confirmed by Sanger sequencing (Fig 1B). Multiplasmic length polymorphisms were observed in the D-loop region and showed a trend for over-representation of cases at the 514 (CA)_n_ and the D16189 loci. **Multiplasmy was observed about 1.5 times more frequently in cases.** Note the tri-allelic multiplasmy results for two subjects (B-76 and S-114) showing 5, 6, and 7 repeat lengths shown in bold. Heteroplasmy/multiplasmy at all these six loci were also confirmed by Sanger sequencing.

*multiplasmy occurred in all brain regions sequenced.

#Val313Ile.

**Fig 2:**
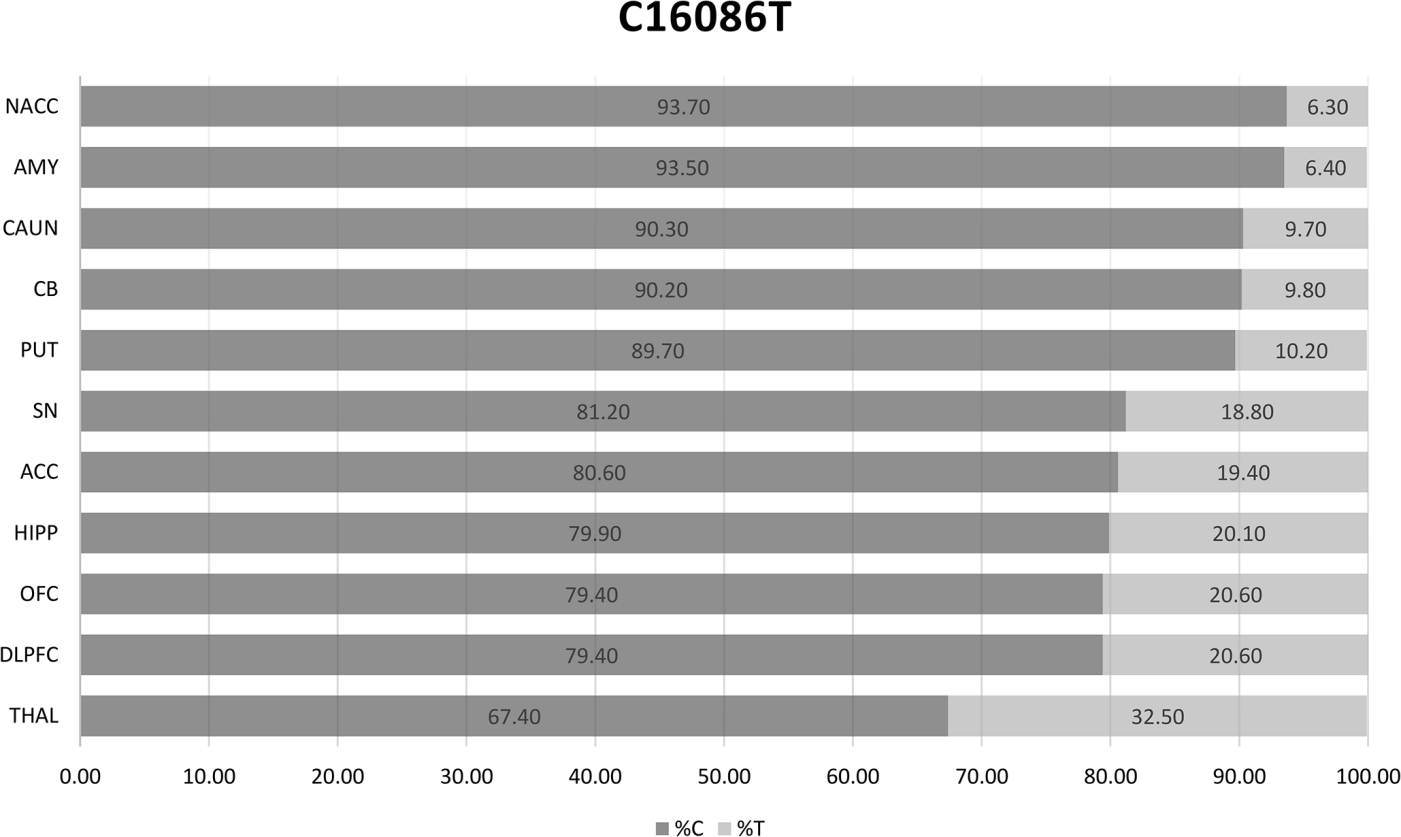
Heteroplasmy levels at position 16086 across 11 brain regions for the same subject.

We further studied the heteroplasmy of T16519C because of the prior association of this SNP in a GAIN/WTCCC2 association analysis in SZ and BD [9]. We confirmed a heteroplasmic T>C substitution at position 16519 of the mitochondrial genome by allele specific PCR using locked nucleic acid primers (LNA-primers) as well as by direct sequencing. Low levels of T16519C heteroplasmy calculated using NGS (~10%) were confirmed by Sanger sequencing as shown in **Fig 1A**. Heteroplasmy levels ranged from 1.74%, 18.9%, and 90% calculated from Illumina NGS results. Interestingly, within the same METH subject, we observed, by NGS and Sanger, homoplasmy in one brain region (100% C in the SN (sample D-84J) and heteroplasmy in the DLPFC (sample D-84E) with 90% C and 10% T (**Fig 1A**). Another example of variable levels of heteroplasmy was observed across all brain regions for a control subject at mt16086 (**Fig 2**). Levels ranged from 6.3% in the NACC to 32.5% in the THAL (**Fig 2**). These findings clearly demonstrate how heteroplasmic mutations can vary between brain regions of the same individual. Interestingly, sample D-84 was a METH user but overall we did not observe an increase in somatic mutations associated with METH.

### Multiplasmy

Multiplasmy is a heteroplasmic variant occurring at a variable length repeat locus. As an example, a repeat of ‘CA’ might be a variable length of 5, 6, and 7 repeats. Thus, a single mtDNA molecule could have one of these three repeat lengths, and taken together in one individual, this locus could have all three repeat lengths. Heteroplasmic deletion/insertion polymorphisms analysis showed a high number of multiplasmic subjects in loci previously known to be particularly hypervariable. Two of these were the poly-cytosine tracts of the hypervariable region, the D310 poly-C tract (CCCCCTCCCCC from position 303 to 316) and the D16189 poly-C tract (CCCCCTCCCC from 16184 to 16193)[29, 30]. Multiplasmy was also observed at a locus known to be a highly variable dinucleotide repeat (CA)_n_ beginning at position 514 of the D-loop. CA_5_ is the rCRS sequence and we observed 4, 5, 6, and 7 CA repeats (**Fig 1B, Table 4**). Multiplasmy was observed about 1.5 times more frequently in cases (**Table 4**). Some subjects (B-76 and S-114) even show tri-allelic multiplasmy with a combination of 5, 6, and 7 CA repeats. The ratio of 514 CA deletions across brain regions was variable (**S2 Fig**), and deletions, when present, were found across all 10 brain regions.

### Blood-brain comparisons

Homoplasmic and heteroplasmic mtDNA variants were compared between blood and 11 brain regions using samples from three control subjects. Although this is too small of a sample to draw any definitive conclusion, there was perfect concordance between the homoplasmic variants found in the 11 brain regions and blood for these three subjects (data not shown), suggesting that blood might be a useful surrogate for the study of homoplasmic mtDNA variation of germ line origin. However, we observed 5 loci with subtle differences for heteroplasmic variants that were present at various levels in brain tissue but undetectable in blood using the 5% cutoff used in the present study (**S8 Table**). Three of these loci (2487, 5755, and 13706) were not present in our database search for reported variants.

## Discussion

We observed several novel and rare mtDNA coding homoplasmic mutations in key genes (ND6, ATP6, CYTB, and ND2). Four novel non-synonymous homoplasmic mutations were validated in different coding regions, three of which were present only in cases and not in controls. There was an excess of non-synonymous homoplasmic mutations found in schizophrenia, but not controls. We also confirmed heteroplasmy at a locus in the D-loop region (T16519C), that we previously reported as being associated with SZ [9]. Excess multiplasmy in cases at the 16189 poly-C tract and at the 514 (CA)_n_ repeat region was also observed, as well as single SZ and BD cases showing striking tri-allelic multiplasmy in brain.

Evidence of genomic instability in the form of somatic variation at heteroplasmic and multiplasmic loci, and novel and rare variants, are particularly interesting in light of recent studies using NGS that showed an excess of novel and rare functional variants in the nuclear genome in different populations [31] and their potential role in complex traits and drug response [32]. A recent study explored the presence of somatic mutations in the aging human brain and showed an accumulation of deletions and single nucleotide variants with age specially in the non-coding hyper-variable region [33], consistent with our findings of somatic heteroplasmic mutations in the adult human brain. The rare and novel coding variants that we found, and the additional non-synonymous mutations in the mtDNA of psychiatric cases, could also support abnormal energy metabolism seen using Magnetic Resonance Spectroscopy (MRS). In general, studies of patients with BD, MDD, and SZ have shown altered energy metabolism in brain [34, 35]. Potential treatment responders to antidepressants sometimes show alterations in MRS profile of energy metabolites [36]. In an animal model of depression there was an altered metabolic profile that was restored to control levels following antidepressant treatment [37]. In view of these evidences, it would be interesting to test the effect of mtDNA variation on energy metabolism in peripheral samples from psychiatric patients.

MtDNA can be methylated [38] suggesting an additional control of mitochondrial transcription and replication. Some of the common T>C and C>T transitions in the hypervariable D-Loop and coding regions are potential methylation sites. The D-loop region heteroplasmic variant T16519C that we previously reported as associated with SZ is a possible candidate for methylation for instance. A few studies have investigated mitochondrial neuroepigenetics [39, 40], and mtDNA epigenetic changes have recently been observed in mammalian brains with age and region specific patterns [41]. Thus, rare and common mitochondria sequence variants while not sufficient to cause a classical mitochondrial disease, may be associated with a cascade involving altered energy output in brain depending on the functional variants and loss or gain of methylation sites in the mtDNA especially in the control region (D-Loop).

We paid particular attention to validation of the observed results and confirmed a subset of variants by Sanger sequencing, allelic specific PCR, and allelic specific PCR using LNA primers. We selected 6 heteroplasmic variants with high levels of heteroplasmy for confirmation by Sanger sequencing, while allele specific methods were needed for levels of heteroplasmy between 10 and 20%. A recent pyrosequencing study of mtDNA in 40 Hapmap reference samples reported high levels of heteroplasmy but low confirmation ratios using only Sanger sequencing, even for high heteroplasmy loci (>40%), raising questions about the efficiency of Sanger to detect heteroplasmy [42]. However, the coverage in that study was lower than ours which might explain why some of the heteroplasmic variants observed were false positives. Additionally, misinterpretation of the chromatograms could also explain some of the observed discordant results, like the G1333A locus that is clearly heteroplasmic but was interpreted as homoplasmic [42].

### Homoplasmic variants

We observed 49 non-synonymous variants at 37 loci that were specific to cases, not found in controls (**Table 1**), 8 of these were predicted using Polyphen as being possibly/probably damaging mutations and could potentially have a functional role in mitochondrial dysfunction and psychiatric disorders. In SZ we observed a particularly high number of non-synonymous mutations per subject (22 variants) (**Table 2**) when compared to non-synonymous variants specific to controls (11 variants). When translated into a Z-score there was an excess of non-synonymous mutations (p = 0.024, two tailed z-score test) in SZ compared to controls. This suggests a higher burden of non-synonymous mutations in SZ and we are conducting new experiments in a larger sample for robust replication. Most of these 49 known non-synonymous variants were not previously associated with any known mitochondrial disorder, ruling out the likelihood that a formal mitochondrial disorder is underlying these psychiatric disorders in this study. However the rate of rare-novel mutations is 5 in 42 psychiatric cases and 2 in 22 controls (**Table 3**), indicating that our sample surpasses the percentages recently reported for mutation rates in screening mitochondrial genomes from symptomatic patients (3%-6%)[43]. We excluded the common haplogroup defining non-synonymous mutations from our calculations, thus we are cautiously optimistic about excess non-synonymous SNPs dispersed across the mtDNA genome and the trend towards an excess in psychiatric disorders particularly in schizophrenia. In the present mtDNA data, the lack of haplogroup specificity for these mutations supports prior literature that has mainly failed to consistently demonstrate differences between major haplogroups in terms of prevalence of psychiatric disorders [4, 5].

### Heteroplasmic Variants

We found novel heteroplasmic loci using NGS. Due to higher sequencing depth and hence higher sensitivity, heteroplasmy as well as somatic mutations are more likely to be detected and reported with NGS as opposed to Sanger sequencing experiments, SNaPshot, Surveyor, etc. [19, 22, 44]. Other technologies such as Sanger sequencing or allelic specific PCR using LNA-primers, must be used to validate heteroplasmy, as we and others find multiple instances of false positives [19, 45].

In general, heteroplasmy can occur in germ-line and become equally distributed throughout many tissues, but it has also been suggested to be a consequence to the effects of reactive oxygen species and other oxidative stress mechanisms inducing substitutions that are not repaired during mtDNA replication [46] or during fission/fusion between mitochondria organelles. On the other hand, recent evidence suggests that somatic mutagenesis is actually influenced by germline mutations that get disseminated by clonal expansion in somatic tissues which can explain also the variable levels of heteroplasmy across the brain and in blood observed in the present study. The present results show equal heteroplasmy in germ-line and brain at some loci, but other loci showed an increase in heteroplasmy in brain with no heteroplasmy found in blood. We report low levels of heteroplasmy in brain tissue not present in blood for three control subjects, underlining the interest in surveying somatic mtDNA variation in brain to uncover mutations possibly involved in neuropsychiatric disorders. Heteroplasmy levels observed in brain exclusively were relatively low usually less than 10% (**S8 Table**), while a perfect concordance of homoplasmic variants between the two tissues was observed.

We confirmed heteroplasmy at T16519C, a locus previously reported as being hypermutable in multiple haplogroups and that we previously found is associated with SZ [9]. Another locus, T16086C, also showed highly variable levels of heteroplasmy (6.3 to 32.5%) between the brain regions from the same control individual (**Fig 2**), suggesting that some brain regions might reach detrimental levels of heteroplasmy.

Many diseases can be caused by heteroplasmic mtDNA mutations with clinical manifestation appearing after a certain threshold of mutant heteroplasmy, a concept called phenotypic threshold effect [47]. Studies have shown heteroplasmy within families and between tissues [48], as well as between cancer and non-cancer tissue from the same individual [22]. Recently, it was shown that heteroplasmy in brain of mice can result in altered metabolic function, as well as altered behavior and cognitive performance [49]. In this study we observed variable levels of heteroplasmy levels between tissues (blood-brain) and within tissue between brain regions from the same individual, pointing to somatic or postzygotic mutations within cells in certain parts of the brain of control subjects. Although no psychiatric cases were assayed for heteroplasmy across brain regions in the present study, we found within controls that heteroplasmic mutations can vary between brain regions from the same individual (**Fig 2**). Low frequency somatic mutations have also been discovered in patients with neurological disorders by whole exome NGS [44]. The authors of the study point to low frequency of mutations in blood as evidence of mosaicism only detectable by high coverage NGS sequencing (>1000X), however as the authors point out they did not have access to brain tissue to determine the distribution of somatic variants associated with the observed neurological alterations [44]. Thus, we will sequence additional brain samples from subjects with psychiatric disorders, to address heteroplasmy across brain regions as a potential indicator of mitochondrial dysfunction.

### Multiplasmy

Multiplasmy was observed in three loci located in the D-loop region (D310, D16189 and 514–523(CA)_n_). Two of these multiplasmy loci consist of poly cytosine tracts interrupted by a thymidine at positions 310 and 16189. These two regions are known to be highly variable and are potentially associated with mitochondria copy number and neurological disorders [29, 50, 51]. Multiplasmy at these loci has been confirmed by cloning and Sanger sequencing [52, 53]. Multiplasmy was also observed at the 514–523 (CA)_n_ locus (**S2 Fig**), a phenomenon previously reported and confirmed by Sanger sequencing [54]. This multiplasmy occurs 1.5 times more frequently in psychiatric cases compared to controls at 514 CA dinucleotide repeat, albeit in a small sample this is not significant (p = 0.14). Multiplasmy in this region can perhaps induce differences in transcription, since this region is at the border of binding of TFAM1 to mtDNA at bp 523–550. We observed variation in multiplasmy levels versus wild type at this locus across brain regions (**S2 Fig**), but multiplasmy was present in all the brain regions examined. The increased multiplasmy in psychiatric brains, while not significant, is perhaps another indicator suggesting an involvement of genetic instability of mtDNA in the predisposition to psychiatric disorders.

In conclusion, we observed several new and rare mitochondrial non-synonymous mutations in psychiatric cases and excess non-synonymous mtDNA mutations in persons with schizophrenia (**Table 1**). These findings support the hypothesis that common and rare mitochondrial mutations can play a role in psychiatric disorders, especially schizophrenia [9, 14–16]. Additionally, in a preliminary analysis, we observed a higher proportion of multiplasmic and heteroplasmic burden primarily in the hyper-variable region in psychiatric cases which was not significantly different from controls. Although speculative, these data point to a higher genomic instability in the mtDNA of psychiatric patients. It is widely known that mtDNA has a higher mutation rate than nuclear DNA, lacks protective histones, and undergoes genomic replication independent of cell division, all of which contribute to functional mutations. Our observations are in agreement with recent reports of an excess of non-synonymous variants in nuclear genes [55] and a higher rate of *de novo* mutations in nuclear genes in schizophrenia [56], as well as somatic mutations present in neurological disorders [44, 57, 58]. A larger study is required in multiple brain regions and blood to directly test our hypothesis that rare and novel mutations in mtDNA are enriched in psychiatric cases.

## Materials and Methods

### Subjects

Anonymized and de-identified post-mortem human brain samples were obtained from the University of California, Irvine Brain Bank (UCIBB: www.vawterlab.com). This study was approved by the Institutional Review Board (IRB) of the University of California, Irvine. Signed informed consent was obtained from next of kin. A total of 14 BD (5F/9M, 54±12yr), 20 controls (5F/15M, 50±18 yr), 15 MDD (8F/7M, 45±12 yr), 14 SZ (7F/7M, 44±9), as well as 6 methamphetamine users with no axis I diagnosis (METH; 4F/2M, 42±8 yr), were analyzed (**S1 and S2 Tables**) resulting in 121 complete mtDNA sequences from 69 subjects. We presented homoplasmic NGS data for 23 of the subjects (cohort 1) in a recent paper [9], in this study we explored also heteroplasmic and multiplasmic mutations in 46 new subjects (cohort 2). In summary, all 69 subjects had the DLPFC sequenced and for a subset of subjects blood as well as other brain regions depending on availability of tissue were also sequenced.

Eleven brain regions were dissected on dry ice from the left hemisphere according to visible landmarks near the regions of interest. DNA was extracted from the following brain regions: anterior cingulate cortex (ACC), amygdala (AMY), caudate nucleus (CAUN), cerebellum (CB), dorsolateral prefrontal cortex (DLPFC), hippocampus (HIPP), nucleus accumbens (NACC), orbitofrontal cortex (OFC), putamen (PUT), substantia nigra (SN), and thalamus (THAL) for 11 subjects and whole blood was also obtained for three of those subjects. DNA was extracted from 25 mg of dissected brain tissue using the DNeasy Blood and Tissue Kit (QIAGEN), according to the manufacturer’s protocol. Previously, DNA was extracted from DLPFC samples (cohort 1) using the phenol phase of a Trizol protocol and precipitated with ethanol [59]. Additional details are available in **S1 Methods**.

### Next-Generation Sequencing Analysis

Mitochondrial NGS was performed as described previously [9]. Briefly, two overlapping mtDNA fragments were PCR amplified, purified, and sequenced using standard manufacturer’s protocols. Reads from Illumina GAII (cohort 1) and HiSeq (cohort 2) were aligned to the mitochondrial revised Cambridge Reference Sequence (rCRS; GenBank accession number NC_012920)[27]. We defined homoplasmic mutations as genetic variants for which the major allele was different from the rCRS and constituted 95% to 100% of the reads. Heteroplasmic mutations were therefore defined as any variant for which the major allele was <95% of the total of the observed alleles. For both homoplasmy and heteroplasmy we filtered out variants with coverage lower than 200 reads. These cutoffs were implemented based on our experience with false positive mutations in problematic regions of the mtDNA. All the mutations reported were successfully confirmed by Sanger sequencing. We focused on results from the DLPFC and SN for reasons of clarity and space (n = 69 unique subjects). We determined whether the mutations were novel or rare by investigating their presence in curated online databases such as MITOMAP containing more than 26,850 Genbank mtDNA sequences [60], mtDB [61], and PhyloTree [28], and also by searching individual genealogical studies reporting mtDNA mutations using Google as suggested [62]. We also compared our sequence variant results to reference databases, 1000 Genomes mitochondrial variants, and to the Phylotree database that contains over 16,500 mtDNA genomes which overlaps MITOMAP. The effect of amino acid substitutions was determined using Polyphen [63, 64].

Quality control was critical especially for heteroplasmic variants. Heteroplasmic A>C variants were observed in many brain samples in the same loci of the mtDNA genome. However, we were unable to confirm these variants using Sanger sequencing or allele specific PCR with locked nucleic acids (**Table 5**). Some of these error hotspots have been previously reported using the Illumina platform [19]. We noted a disproportionate number of A>C heteroplasmic substitutions (148 out of 401 heteroplasmic variants) which was more than what would be expected, as transversions occur less frequently than transitions. Two previously reported error hotspots in Illumina NGS [19], the A3492C and A10306C loci, and a transversion never reported at position A6419C were frequently observed (**Table 5**). Three other non A/C heteroplasmic positions that did not validate were T3488A, T6415A, and G9801T. Closer examination of the sequence surrounding the A>C sequencing error hotspots revealed a common motif of at least two A nucleotides followed by at least two C nucleotides (**Table 5**). We choose for validation 6 of the heteroplasmic variants with heteroplasmy levels of more than 10% to be confirmed by Sanger sequencing and they were all successfully confirmed.

**Table 5:** Sequencing error hotspots in mtDNA.

Position	Context	Variant	N (samples)#
302	acc**aaAccc**cc	A/C	47
3492	cta**aaAccc**gc	A/C	106
6419	ata**aaAccc**cc	A/C	109
10306	aacta**A**cctgc	A/C	41

Four variant loci not previously reported showed heteroplasmic A/C transversions in a large number of samples that could not be validated by Sanger sequencing or by allele specific PCR using LNA-primers.

^**#**^ 121 samples were sequenced (S2 Table), this column shows the number of samples with the aberrant mutations that were errors.

## Supporting Information

S1 Fig Phylogenetic tree of mtDNA variants observed in the present cohort. The first letter of the subject label corresponds to the diagnosis (B: bipolar disorder; S: schizophrenia; M: major depression; D: drugs (methamphetamine)), the number is the age of the subject, and the letter after the age is the gender (F: female; M: male).
(DOCX)

S2 Fig The ratio of the deletion to wild type at 514 CA across brain regions. Note for both subjects that the ratio is quite variable from 1.35–3.24, while the other subject is 1.57–2.73.
(DOCX)

S1 Methods (DOCX)

S1 Table Subject demographics by NGS platform. (DOCX)

S2 Table Number of samples processed in brain and blood for next generation sequencing. (DOCX)

S3 Table Distribution of the 984 homoplasmic SNPs (compared to the rCRS) observed in 65 DLPFC samples sequenced (two DLPFC were from METH subjects). (DOCX)

S4 Table Total number of non-synonymous mtDNA homoplasmic sequence substitutions in 65 DLPFC samples. (DOCX)

S5 Table The distribution of subjects by number of non-synonymous coding mutations categorized by diagnosis. There was a non-significant excess of 5+ mutations in cases compared with controls (Fishers Exact Test, one-sided p = 0.068).
(DOCX)

S6 Table Homoplasmic mutations in 12S and 16S rRNA genes. In gray are the variants only present in cases but not in controls.
(DOCX)

S7 Table Heteroplasmic variants in the DLPFC were mainly clustered in the D-Loop region. (DOCX)

S8 Table Heteroplasmic variants that were present at various levels in brain tissue but undetectable in blood. (DOCX)

## References

[1] Rezin GT, Amboni G, Zugno AI, Quevedo J, Streck EL. Mitochondrial dysfunction and psychiatric disorders. Neurochem Res. 2009;34(6):1021–9. Epub 2008/11/04. 10.1007/s11064-008-9865-8 . 18979198

[2] Verge B, Alonso Y, Miralles C, Valero J, Vilella E, Boles RG, et al New evidence for the involvement of mitochondrial inheritance in schizophrenia: results from a cross-sectional study evaluating the risk of illness in relatives of schizophrenia patients. J Clin Psychiatry. 2012;73(5):684–90. Epub 2012/04/07. 10.4088/JCP.10m06718 . 22480934

[3] Mosquera-Miguel A, Torrell H, Abasolo N, Arrojo M, Paz E, Ramos-Rios R, et al. No evidence that major mtDNA European haplogroups confer risk to schizophrenia. Am J Med Genet B Neuropsychiatr Genet. 2012. Epub 2012/04/03. 10.1002/ajmg.b.32044 . 22467472

[4] McMahon FJ, Chen YS, Patel S, Kokoszka J, Brown MD, Torroni A, et al Mitochondrial DNA sequence diversity in bipolar affective disorder. Am J Psychiatry. 2000;157(7):1058–64. Epub 2000/06/30. . 10873911 10.1176/appi.ajp.157.7.1058

[5] Bertolin C, Magri C, Barlati S, Vettori A, Perini GI, Peruzzi P, et al Analysis of complete mitochondrial genomes of patients with schizophrenia and bipolar disorder. J Hum Genet. 2011;56(12):869–72. Epub 2011/10/14. 10.1038/jhg.2011.111 . 21993419

[6] Kazuno AA, Munakata K, Mori K, Nanko S, Kunugi H, Nakamura K, et al Mitochondrial DNA haplogroup analysis in patients with bipolar disorder. Am J Med Genet B Neuropsychiatr Genet. 2009;150B(2):243–7. Epub 2008/06/12. 10.1002/ajmg.b.30804 . 18546119

[7] Wang GX, Zhang Y, Zhang YT, Dong YS, Lv ZW, Sun M, et al Mitochondrial haplogroups and hypervariable region polymorphisms in schizophrenia: a case-control study. Psychiatry Res. 2013;209(3):279–83. Epub 2013/02/05. 10.1016/j.psychres.2013.01.001 . 23374981

[8] Anglin RE, Mazurek MF, Tarnopolsky MA, Rosebush PI. The mitochondrial genome and psychiatric illness. Am J Med Genet B Neuropsychiatr Genet. 2012;159B(7):749–59. Epub 2012/08/14. 10.1002/ajmg.b.32086 . 22887963

[9] Sequeira A, Martin MV, Rollins B, Moon EA, Bunney WE, Macciardi F, et al Mitochondrial mutations and polymorphisms in psychiatric disorders. Front Genet. 2012;3:103 Epub 2012/06/23. 10.3389/fgene.2012.00103 22723804 PMC3379031

[10] Verge B, Alonso Y, Valero J, Miralles C, Vilella E, Martorell L. Mitochondrial DNA (mtDNA) and schizophrenia. Eur Psychiatry. 2011;26(1):45–56. Epub 2010/10/29. 10.1016/j.eurpsy.2010.08.008 . 20980130

[11] Kato M, Nakamura M, Ichiba M, Tomiyasu A, Shimo H, Higuchi I, et al Mitochondrial DNA deletion mutations in patients with neuropsychiatric symptoms. Neurosci Res. 2011;69(4):331–6. Epub 2010/12/28. 10.1016/j.neures.2010.12.013 . 21185889

[12] Rollins B, Martin MV, Sequeira PA, Moon EA, Morgan LZ, Watson SJ, et al Mitochondrial variants in schizophrenia, bipolar disorder, and major depressive disorder. PLoS One. 2009;4(3):e4913 Epub 2009/03/18. 10.1371/journal.pone.0004913 19290059 PMC2654519

[13] Hudson G, Gomez-Duran A, Wilson IJ, Chinnery PF. Recent mitochondrial DNA mutations increase the risk of developing common late-onset human diseases. PLoS Genet. 2014;10(5):e1004369 Epub 2014/05/24. 10.1371/journal.pgen.1004369 . 24852434 PMC4031051

[14] Inczedy-Farkas G, Remenyi V, Gal A, Varga Z, Balla P, Udvardy-Meszaros A, et al Psychiatric symptoms of patients with primary mitochondrial DNA disorders. Behav Brain Funct. 2012;8:9 Epub 2012/02/15. 10.1186/1744-9081-8-9 22329956 PMC3348039

[15] Anglin RE, Garside SL, Tarnopolsky MA, Mazurek MF, Rosebush PI. The psychiatric manifestations of mitochondrial disorders: a case and review of the literature. J Clin Psychiatry. 2012;73(4):506–12. Epub 2012/05/15. 10.4088/JCP.11r07237 . 22579150

[16] Anglin RE, Tarnopolsky MA, Mazurek MF, Rosebush PI. The psychiatric presentation of mitochondrial disorders in adults. J Neuropsychiatry Clin Neurosci. 2012;24(4):394–409. Epub 2012/12/12. 10.1176/appi.neuropsych.11110345 . 23224446

[17] Iborra FJ, Kimura H, Cook PR. The functional organization of mitochondrial genomes in human cells. BMC Biol. 2004;2:9 Epub 2004/05/26. 10.1186/1741-7007-2-9 15157274 PMC425603

[18] Satoh M, Kuroiwa T. Organization of multiple nucleoids and DNA molecules in mitochondria of a human cell. Exp Cell Res. 1991;196(1):137–40. Epub 1991/09/01. . 1715276 10.1016/0014-4827(91)90467-9

[19] Li M, Schonberg A, Schaefer M, Schroeder R, Nasidze I, Stoneking M. Detecting heteroplasmy from high-throughput sequencing of complete human mitochondrial DNA genomes. Am J Hum Genet. 2010;87(2):237–49. Epub 2010/08/11. 10.1016/j.ajhg.2010.07.014 20696290 PMC2917713

[20] Huang T. Next generation sequencing to characterize mitochondrial genomic DNA heteroplasmy. Curr Protoc Hum Genet. 2011;Chapter 19:Unit19 8. Epub 2011/10/07. 10.1002/0471142905.hg1908s71 . 21975941 PMC4687495

[21] Yamamoto BK, Raudensky J. The role of oxidative stress, metabolic compromise, and inflammation in neuronal injury produced by amphetamine-related drugs of abuse. J Neuroimmune Pharmacol. 2008;3(4):203–17. Epub 2008/08/19. 10.1007/s11481-008-9121-7 18709468 PMC3955110

[22] He Y, Wu J, Dressman DC, Iacobuzio-Donahue C, Markowitz SD, Velculescu VE, et al Heteroplasmic mitochondrial DNA mutations in normal and tumour cells. Nature. 2010;464(7288):610–4. Epub 2010/03/05. 10.1038/nature08802 20200521 PMC3176451

[23] Zaragoza MV, Fass J, Diegoli M, Lin D, Arbustini E. Mitochondrial DNA variant discovery and evaluation in human Cardiomyopathies through next-generation sequencing. PLoS One. 2010;5(8):e12295 Epub 2010/09/03. 10.1371/journal.pone.0012295 20808834 PMC2924892

[24] Sondheimer N, Glatz CE, Tirone JE, Deardorff MA, Krieger AM, Hakonarson H. Neutral mitochondrial heteroplasmy and the influence of aging. Hum Mol Genet. 2011;20(8):1653–9. Epub 2011/02/08. 10.1093/hmg/ddr043 21296868 PMC3063991

[25] Guo Y, Cai Q, Samuels DC, Ye F, Long J, Li CI, et al The use of next generation sequencing technology to study the effect of radiation therapy on mitochondrial DNA mutation. Mutat Res. 2012;744(2):154–60. Epub 2012/03/06. 10.1016/j.mrgentox.2012.02.006 22387842 PMC3354959

[26] Belle EM, Piganeau G, Gardner M, Eyre-Walker A. An investigation of the variation in the transition bias among various animal mitochondrial DNA. Gene. 2005;355:58–66. Epub 2005/07/26. 10.1016/j.gene.2005.05.019 . 16039074

[27] Andrews RM, Kubacka I, Chinnery PF, Lightowlers RN, Turnbull DM, Howell N. Reanalysis and revision of the Cambridge reference sequence for human mitochondrial DNA. Nat Genet. 1999;23(2):147 . 10508508 10.1038/13779

[28] van Oven M, Kayser M. Updated comprehensive phylogenetic tree of global human mitochondrial DNA variation. Hum Mutat. 2009;30(2):E386–94. Epub 2008/10/15. 10.1002/humu.20921 . 18853457

[29] Liou CW, Lin TK, Chen JB, Tiao MM, Weng SW, Chen SD, et al Association between a common mitochondrial DNA D-loop polycytosine variant and alteration of mitochondrial copy number in human peripheral blood cells. J Med Genet. 2010;47(11):723–8. Epub 2010/09/15. 10.1136/jmg.2010.077552 . 20837494

[30] Tang M, Baez S, Pruyas M, Diaz A, Calvo A, Riquelme E, et al Mitochondrial DNA mutation at the D310 (displacement loop) mononucleotide sequence in the pathogenesis of gallbladder carcinoma. Clin Cancer Res. 2004;10(3):1041–6. Epub 2004/02/12. . 14871983 10.1158/1078-0432.ccr-0701-3

[31] Tennessen JA, Bigham AW, O'Connor TD, Fu W, Kenny EE, Gravel S, et al Evolution and functional impact of rare coding variation from deep sequencing of human exomes. Science. 2012;337(6090):64–9. Epub 2012/05/19. 10.1126/science.1219240 22604720 PMC3708544

[32] Nelson MR, Wegmann D, Ehm MG, Kessner D, St Jean P, Verzilli C, et al An abundance of rare functional variants in 202 drug target genes sequenced in 14,002 people. Science. 2012;337(6090):100–4. Epub 2012/05/19. 10.1126/science.1217876 . 22604722 PMC4319976

[33] Williams SL, Mash DC, Zuchner S, Moraes CT. Somatic mtDNA mutation spectra in the aging human putamen. PLoS Genet. 2013;9(12):e1003990 Epub 2013/12/18. 10.1371/journal.pgen.1003990 24339796 PMC3854840

[34] Gruber S, Frey R, Mlynarik V, Stadlbauer A, Heiden A, Kasper S, et al Quantification of metabolic differences in the frontal brain of depressive patients and controls obtained by 1H-MRS at 3 Tesla. Invest Radiol. 2003;38(7):403–8. Epub 2003/06/25. 10.1097/01.rli.0000073446.43445.20 . 12821853

[35] Ongur D, Prescot AP, Jensen JE, Cohen BM, Renshaw PF. Creatine abnormalities in schizophrenia and bipolar disorder. Psychiatry Res. 2009;172(1):44–8. Epub 2009/02/26. 10.1016/j.pscychresns.2008.06.002 19239984 PMC2729651

[36] Caverzasi E, Pichiecchio A, Poloni GU, Calligaro A, Pasin M, Palesi F, et al Magnetic resonance spectroscopy in the evaluation of treatment efficacy in unipolar major depressive disorder: a review of the literature. Funct Neurol. 2012;27(1):13–22. Epub 2012/06/13. . 22687162 PMC3812759

[37] Kim SY, Lee YJ, Kim H, Lee DW, Woo DC, Choi CB, et al Desipramine attenuates forced swim test-induced behavioral and neurochemical alterations in mice: an in vivo(1)H-MRS study at 9.4T. Brain Res. 2010;1348:105–13. Epub 2010/06/15. 10.1016/j.brainres.2010.05.097 . 20542016

[38] Bellizzi D, D'Aquila P, Scafone T, Giordano M, Riso V, Riccio A, et al. The Control Region of Mitochondrial DNA Shows an Unusual CpG and Non-CpG Methylation Pattern. DNA Res. 2013. Epub 2013/06/28. 10.1093/dnares/dst029 . 23804556 PMC3859322

[39] Manev H, Dzitoyeva S, Chen H. Mitochondrial DNA: A Blind Spot in Neuroepigenetics. Biomol Concepts. 2012;3(2):107–15. Epub 2012/05/29. 10.1515/bmc-2011-0058 22639700 PMC3359012

[40] Shock LS, Thakkar PV, Peterson EJ, Moran RG, Taylor SM. DNA methyltransferase 1, cytosine methylation, and cytosine hydroxymethylation in mammalian mitochondria. Proc Natl Acad Sci U S A. 2011;108(9):3630–5. Epub 2011/02/16. 10.1073/pnas.1012311108 21321201 PMC3048134

[41] Dzitoyeva S, Chen H, Manev H. Effect of aging on 5-hydroxymethylcytosine in brain mitochondria. Neurobiol Aging. 2012;33(12):2881–91. Epub 2012/03/27. 10.1016/j.neurobiolaging.2012.02.006 22445327 PMC3462297

[42] Sosa MX, Sivakumar IK, Maragh S, Veeramachaneni V, Hariharan R, Parulekar M, et al Next-generation sequencing of human mitochondrial reference genomes uncovers high heteroplasmy frequency. PLoS Comput Biol. 2012;8(10):e1002737 Epub 2012/11/08. 10.1371/journal.pcbi.1002737 23133345 PMC3486893

[43] Tang S, Wang J, Zhang VW, Li FY, Landsverk M, Cui H, et al Transition to next generation analysis of the whole mitochondrial genome: a summary of molecular defects. Hum Mutat. 2013;34(6):882–93. Epub 2013/03/07. 10.1002/humu.22307 . 23463613

[44] Jamuar SS, Lam AT, Kircher M, D'Gama AM, Wang J, Barry BJ, et al Somatic mutations in cerebral cortical malformations. N Engl J Med. 2014;371(8):733–43. Epub 2014/08/21. 10.1056/NEJMoa1314432 . 25140959 PMC4274952

[45] Bandelt HJ, Salas A. Current next generation sequencing technology may not meet forensic standards. Forensic Sci Int Genet. 2012;6(1):143–5. Epub 2011/05/14. 10.1016/j.fsigen.2011.04.004 . 21565569

[46] Wallace DC. Bioenergetic origins of complexity and disease. Cold Spring Harb Symp Quant Biol. 2011;76:1–16. Epub 2011/12/24. 10.1101/sqb.2011.76.010462 . 22194359 PMC4405153

[47] Rossignol R, Faustin B, Rocher C, Malgat M, Mazat JP, Letellier T. Mitochondrial threshold effects. Biochem J. 2003;370(Pt 3):751–62. Epub 2002/12/07. 10.1042/bj20021594 12467494 PMC1223225

[48] Goto H, Dickins B, Afgan E, Paul IM, Taylor J, Makova KD, et al Dynamics of mitochondrial heteroplasmy in three families investigated via a repeatable re-sequencing study. Genome Biol. 2011;12(6):R59 Epub 2011/06/28. 10.1186/gb-2011-12-6-r59 21699709 PMC3218847

[49] Sharpley MS, Marciniak C, Eckel-Mahan K, McManus M, Crimi M, Waymire K, et al Heteroplasmy of mouse mtDNA is genetically unstable and results in altered behavior and cognition. Cell. 2012;151(2):333–43. Epub 2012/10/16. 10.1016/j.cell.2012.09.004 . 23063123 PMC4175720

[50] Coto E, Gomez J, Alonso B, Corao AI, Diaz M, Menendez M, et al Late-onset Alzheimer's disease is associated with mitochondrial DNA 7028C/haplogroup H and D310 poly-C tract heteroplasmy. Neurogenetics. 2011;12(4):345–6. Epub 2011/08/09. 10.1007/s10048-011-0295-4 . 21822896

[51] Wang PN, Lee HC, Wang CH, Ping YH, Liu TY, Chi CW, et al Heteroplasmy of mitochondrial D310 mononucleotide repeat region in the blood of patients with Alzheimer's disease. J Alzheimers Dis. 2009;18(2):345–53. Epub 2009/07/09. 10.3233/jad-2009-1156 . 19584455

[52] Lutz-Bonengel S, Sanger T, Pollak S, Szibor R. Different methods to determine length heteroplasmy within the mitochondrial control region. Int J Legal Med. 2004;118(5):274–81. Epub 2004/05/26. 10.1007/s00414-004-0457-0 . 15160269

[53] Howell N, Smejkal CB. Persistent heteroplasmy of a mutation in the human mtDNA control region: hypermutation as an apparent consequence of simple-repeat expansion/contraction. Am J Hum Genet. 2000;66(5):1589–98. Epub 2000/04/14. 10.1086/302910 10762545 PMC1378018

[54] Forster L, Forster P, Gurney SM, Spencer M, Huang C, Rohl A, et al Evaluating length heteroplasmy in the human mitochondrial DNA control region. Int J Legal Med. 2010;124(2):133–42. Epub 2009/11/26. 10.1007/s00414-009-0385-0 . 19937256

[55] Xu B, Roos JL, Dexheimer P, Boone B, Plummer B, Levy S, et al Exome sequencing supports a de novo mutational paradigm for schizophrenia. Nat Genet. 2011;43(9):864–8. Epub 2011/08/09. 10.1038/ng.902 21822266 PMC3196550

[56] Girard SL, Gauthier J, Noreau A, Xiong L, Zhou S, Jouan L, et al Increased exonic de novo mutation rate in individuals with schizophrenia. Nat Genet. 2011;43(9):860–3. Epub 2011/07/12. 10.1038/ng.886 . 21743468

[57] Poduri A, Evrony GD, Cai X, Walsh CA. Somatic mutation, genomic variation, and neurological disease. Science. 2013;341(6141):1237758 Epub 2013/07/06. 10.1126/science.1237758 23828942 PMC3909954

[58] Hu WF, Chahrour MH, Walsh CA. The diverse genetic landscape of neurodevelopmental disorders. Annu Rev Genomics Hum Genet. 2014;15:195–213. Epub 2014/09/04. 10.1146/annurev-genom-090413-025600 . 25184530 PMC10591257

[59] Shao L, Martin MV, Watson SJ, Schatzberg A, Akil H, Myers RM, et al Mitochondrial involvement in psychiatric disorders. Ann Med. 2008;40(4):281–95. 10.1080/07853890801923753 18428021 PMC3098560

[60] Ruiz-Pesini E, Lott MT, Procaccio V, Poole JC, Brandon MC, Mishmar D, et al An enhanced MITOMAP with a global mtDNA mutational phylogeny. Nucleic Acids Res. 2007;35(Database issue):D823–8. Epub 2006/12/21. 10.1093/nar/gkl927 17178747 PMC1781213

[61] Ingman M, Gyllensten U. mtDB: Human Mitochondrial Genome Database, a resource for population genetics and medical sciences. Nucleic Acids Research. 2006;34(Database issue):D749–51. Epub 2005/12/31. 10.1093/nar/gkj010 16381973 PMC1347373

[62] Bandelt HJ, Salas A, Taylor RW, Yao YG. Exaggerated status of "novel" and "pathogenic" mtDNA sequence variants due to inadequate database searches. Hum Mutat. 2009;30(2):191–6. Epub 2008/09/19. 10.1002/humu.20846 . 18800376

[63] Ramensky V, Bork P, Sunyaev S. Human non-synonymous SNPs: server and survey. Nucleic Acids Res. 2002;30(17):3894–900. Epub 2002/08/31. 12202775 10.1093/nar/gkf493 PMC137415

[64] Adzhubei IA, Schmidt S, Peshkin L, Ramensky VE, Gerasimova A, Bork P, et al A method and server for predicting damaging missense mutations. Nat Methods. 2010;7(4):248–9. Epub 2010/04/01. 10.1038/nmeth0410-248 20354512 PMC2855889

