# Supplementary material for: Mitochondrial Mutations in Subjects with Psychiatric Disorders
Source: PLoS One. 2015 May 26;10(5):e0127280. doi: 10.1371/journal.pone.0127280 (PMC4444211; doi:10.1371/journal.pone.0127280)
Supplement: S1 Fig — The first letter of the subject label corresponds to the diagnosis (B: bipolar disorder; S: schizophrenia; M: major depression; D: drugs (methamphetamine)), the number is the age of the subject, and the letter after the age is the gender (F: female; M: male). (DOCX) [file pone.0127280.s001.docx]

**S1 Fig**. Phylogenetic tree of mtDNA variants observed in the present cohort.
