# Supplementary material for: Mitochondrial Mutations in Subjects with Psychiatric Disorders
Source: PLoS One. 2015 May 26;10(5):e0127280. doi: 10.1371/journal.pone.0127280 (PMC4444211; doi:10.1371/journal.pone.0127280)
Supplement: S2 Fig — Note for both subjects that the ratio is quite variable from 1.35–3.24, while the other subject is 1.57–2.73. (DOCX) [file pone.0127280.s002.docx]

**S2 Fig.** The ratio of the deletion to wild type at 514 CA across brain regions. Note for both subjects that the ratio is quite variable from 1.35 - 3.24, while the other subject is 1.57 - 2.73.
