# Supplementary material for: Mitochondrial Mutations in Subjects with Psychiatric Disorders
Source: PLoS One. 2015 May 26;10(5):e0127280. doi: 10.1371/journal.pone.0127280 (PMC4444211; doi:10.1371/journal.pone.0127280)
Supplement: S1 Methods — (DOCX) [file pone.0127280.s003.docx]

**S1 Methods.**The human brain dissection and freezing protocol is described in detail elsewhere (Jones et al. 1992). Briefly, after collection coronal brain sections were flash frozen using liquid nitrogen vapor. The brains were then stored at -80°C until further dissection. Eleven brain regions from 5 control subjects were dissected on dry ice from the left hemisphere according to visible landmarks near the regions of interest. DNA was extracted from the following brain regions: anterior cingulate cortex (ACC), amygdala (AMY), caudate nucleus (CAUN), cerebellum (CB), dorsolateral prefrontal cortex (DLPFC), hippocampus (HIPP), nucleus accumbens (NACC), orbitofrontal cortex (OFC), putamen (PUT), substantia nigra (SN), and thalamus (THAL). In addition to brain tissue, whole blood was available for 3 of these 5 subjects. Finally, DLPFC only was extracted from 18 additional subjects (cohort 1) **(S2 Table)**.

DNA was extracted from 25 mg brain samples using the DNeasy Blood and Tissue Kit (QIAGEN), according to the manufacturer’s protocol. Genomic DNA for the DLPFC was previously extracted for certain subjects (N= 23) ([Sequeira et al., 2012](#_ENREF_35)). There is no overlap in mutation analysis performed in our prior paper ([Sequeira et al., 2012](#_ENREF_35)) and this study. We doubled the original sample by another 46 subjects (cohort 2) **(S1 Table)**, to a total of 69 unique subjects in this study. In total, we analyzed whole mtDNA genome sequence data from 121 samples (65 DLPFC, 3 blood, and 53 additional brain regions including the SN from 6 METH subjects). The clinical phenotype was established by a team of mental health professionals as described previously ([Sequeira et al., 2012](#_ENREF_35)). Sanger sequencing and allele specific PCR using locked nucleic acids (LNA) were used to confirm variants and to identify error hotspots.
