# Supplementary material for: Mitochondrial Mutations in Subjects with Psychiatric Disorders
Source: PLoS One. 2015 May 26;10(5):e0127280. doi: 10.1371/journal.pone.0127280 (PMC4444211; doi:10.1371/journal.pone.0127280)
Supplement: S1 Table — (DOCX) [file pone.0127280.s004.docx]

**S1 Table.** Subject demographics by NGS platform. No statistical differences were found between diagnoses in terms of age (BD=54±12; C=50±19; MDD=45±12; SZ=44±9; Meth=42±8).

| Subject | Age | Gender | Diagnosis | Haplogroup | Platform |
| --- | --- | --- | --- | --- | --- |
| B-10 | 66 | F | BD | X2c1b | GAII |
| B-11 | 52 | M | BD | H1 | GAII |
| B-15 | 63 | M | BD | H10e | GAII |
| B-22 | 49 | M | BD | X2 | GAII |
| B-71 | 70 | F | BD | X2a2 | HiSeq |
| B-72 | 39 | M | BD | HV9a1 | HiSeq |
| B-73 | 62 | M | BD | H48 | HiSeq |
| B-74 | 74 | F | BD | H10a | HiSeq |
| B-75 | 59 | F | BD | T2b4 | HiSeq |
| B-76 | 39 | M | BD | I3b | HiSeq |
| B-77 | 50 | M | BD | H1b | HiSeq |
| B-78 | 50 | M | BD | A2d | HiSeq |
| B-79 | 43 | M | BD | H1 | HiSeq |
| B-80 | 38 | F | BD | J1c2o | HiSeq |
| C-1 | 40 | M | C | H1e4a | GAII |
| C-2 | 18 | M | C | X2c1 | GAII |
| C-25 | 64 | M | C | H5d | GAII |
| C-4 | 55 | M | C | H1 | GAII |
| C-5 | 50 | M | C | H2a2a1a | GAII |
| C-6 | 54 | M | C | I1 | GAII |
| C-36 | 53 | F | C | H26a1 | GAII |
| C-47 | 28 | M | C | U2e2a3 | GAII |
| C-58 | 58 | F | C | L1c3b2 | GAII |
| C-69 | 103 | F | C | HV1a2 | GAII |
| C-81 | 34 | F | C | I2 | HiSeq |
| C-82 | 48 | M | C | H1c3 | HiSeq |
| C-83 | 44 | M | C | U5b1e1 | HiSeq |
| C-87 | 35 | M | C | D1d | HiSeq |
| C-88 | 57 | M | C | U5a1b3 | HiSeq |
| C-89 | 68 | M | C | H1ap1 | HiSeq |
| C-90 | 32 | F | C | B2g | HiSeq |
| C-91 | 67 | M | C | U5a1b | HiSeq |
| C-92 | 52 | M | C | H3g3 | HiSeq |
| C-93 | 43 | M | C | T2b | HiSeq |
| M-3 | 19 | M | MDD | D1f | GAII |
| M-7 | 52 | M | MDD | X2c1 | GAII |
| M-13 | 49 | M | MDD | U5a1d2b | GAII |
| M-18 | 35 | M | MDD | N1a1a1a2 | GAII |
| M-20 | 50 | F | MDD | I2d | GAII |
| M-94 | 53 | F | MDD | H1e2 | HiSeq |
| M-95 | 44 | F | MDD | U5a1a1d | HiSeq |
| M-96 | 61 | M | MDD | I4a | HiSeq |
| M-97 | 63 | F | MDD | H1b1 | HiSeq |
| M-98 | 41 | F | MDD | HV | HiSeq |
| M-99 | 42 | F | MDD | K2a10 | HiSeq |
| M-100 | 41 | F | MDD | V16 | HiSeq |
| M-101 | 24 | F | MDD | T2b | HiSeq |
| M-102 | 50 | M | MDD | H61 | HiSeq |
| M-103 | 54 | M | MDD | K1a3a | HiSeq |
| S-8 | 45 | M | SZ | H1g1 | GAII |
| S-9 | 53 | F | SZ | T1a1a1 | GAII |
| S-12 | 32 | F | SZ | U5a1 | GAII |
| S-21 | 36 | M | SZ | U2e1c | GAII |
| S-105 | 47 | F | SZ | H1b1 | HiSeq |
| S-106 | 59 | M | SZ | H4a1a1a | HiSeq |
| S-107 | 45 | M | SZ | H2a2a1 | HiSeq |
| S-108 | 62 | F | SZ | V | HiSeq |
| S-109 | 35 | F | SZ | H3h | HiSeq |
| S-110 | 41 | F | SZ | U5b2a1a1b | HiSeq |
| S-111 | 47 | M | SZ | H56 | HiSeq |
| S-112 | 35 | M | SZ | A2p | HiSeq |
| S-113 | 45 | M | SZ | H1ag | HiSeq |
| S-114 | 40 | F | SZ | K1b2b | HiSeq |
| D-70 | 49 | F | Meth | H7c | HiSeq |
| D-84 | 34 | F | Meth | H69 | HiSeq |
| D-85 | 35 | M | Meth | A2c | HiSeq |
| D-86 | 39 | F | Meth | H2a2b1a | HiSeq |
| D-104 | 40 | F | Meth | H1 | HiSeq |
| D-115 | 55 | M | Meth | V3a1 | HiSeq |
