# Supplementary material for: Mitochondrial Mutations in Subjects with Psychiatric Disorders
Source: PLoS One. 2015 May 26;10(5):e0127280. doi: 10.1371/journal.pone.0127280 (PMC4444211; doi:10.1371/journal.pone.0127280)
Supplement: S2 Table — (DOCX) [file pone.0127280.s005.docx]

**S2 Table.** Number of samples processed in brain and blood for next generation sequencing.

| **Cohort** | **Subjects** | **Samples** | **DLPFC** | **Blood** | **Other Brain Regions** |
| --- | --- | --- | --- | --- | --- |
| 1 | 23 | 73 | 23 | 3 | 47 |
| 2 | 46 | 48 | 42 | 0 | 6 |
| Total | 69 | 121 | 65 | 3 | 53 |
