# Supplementary material for: Mitochondrial Mutations in Subjects with Psychiatric Disorders
Source: PLoS One. 2015 May 26;10(5):e0127280. doi: 10.1371/journal.pone.0127280 (PMC4444211; doi:10.1371/journal.pone.0127280)
Supplement: S3 Table — (DOCX) [file pone.0127280.s006.docx]

**S3 Table**. Distribution of the 984 homoplasmic SNPs (compared to the rCRS) observed in 65 DLPFC samples sequenced (two DLPFC were from METH subjects).

| **Annotations** | **Ctrl (20)** | **BD (14)** | **MDD (15)** | **SCZ (14)** | **Meth (2)** | **Total** |
| --- | --- | --- | --- | --- | --- | --- |
| ATP6 | 10 | 8 | 6 | 5 |  | 29 |
| ATP8 | 6 | 1 | 3 |  |  | 10 |
| COX1 | 21 | 10 | 3 | 10 |  | 44 |
| COX2 | 8 | 3 | 2 | 5 |  | 18 |
| COX3 | 10 | 3 | 6 | 7 |  | 26 |
| CYTB | 23 | 8 | 22 | 15 |  | 68 |
| ND1 | 10 | 5 | 7 | 11 |  | 33 |
| ND2 | 16 | 9 | 13 | 13 |  | 51 |
| ND3 | 8 | 4 | 9 | 3 |  | 24 |
| ND4 | 18 | 7 | 14 | 10 |  | 49 |
| ND4L | 3 |  | 3 | 2 |  | 8 |
| ND5 | 36 | 15 | 22 | 17 |  | 90 |
| ND6 | 7 | 8 | 5 | 8 |  | 28 |
| **Polypeptides** | **176** | **81** | **115** | **106** | **0** | **478** |
| 12SrRNA | 6 | 4 | 6 | 5 |  | 21 |
| 16SrRNA | 18 | 14 | 17 | 11 | 1 | 61 |
| **rRNAs** | **24** | **18** | **23** | **16** | **1** | **82** |
| TRNA |  |  |  | 1 |  | 1 |
| TRNC | 2 |  | 1 |  |  | 3 |
| TRND | 1 |  | 1 |  |  | 2 |
| TRNE |  |  | 1 |  |  | 1 |
| TRNG | 2 | 1 | 2 | 2 |  | 7 |
| TRNL2 | 5 |  | 4 | 4 |  | 13 |
| TRNN | 1 |  |  |  |  | 1 |
| TRNP | 1 |  |  |  |  | 1 |
| TRNR | 1 | 1 | 1 | 1 |  | 4 |
| TRNT | 6 | 3 | 5 | 3 |  | 17 |
| TRNW |  |  |  | 1 |  | 1 |
| **TRNs** | **19** | **5** | **15** | **12** | **0** | **51** |
| D-Loop | 123 | 83 | 94 | 68 | 2 | 370 |
| **Non-coding nucleotides** | 2 |  | 1 |  |  | 3 |
| **Grand Total** | **344** | **187** | **248** | **202** | **3** | **984** |
