# Supplementary material for: Mitochondrial Mutations in Subjects with Psychiatric Disorders
Source: PLoS One. 2015 May 26;10(5):e0127280. doi: 10.1371/journal.pone.0127280 (PMC4444211; doi:10.1371/journal.pone.0127280)
Supplement: S4 Table — (DOCX) [file pone.0127280.s007.docx]

**S4 Table**. Total number of non-synonymous mtDNA homoplasmic sequence substitutions in 65 DLPFC samples.

| **Gene** | **Ctrl (20)** | **BD (14)** | **MDD (15)** | **SCZ (14)** | **Meth (2)** | **Total** |
| --- | --- | --- | --- | --- | --- | --- |
| **ATP6** | **6** | **5** | **4** | **2** | **0** | **17** |
| **ATP8** | **1** | **1** | **2** | **0** | **0** | **4** |
| **COX1** | **1** | **1** | **1** | **1** | **0** | **4** |
| **COX2** | **1** | **2** |  | **1** | **0** | **4** |
| **COX3** | **6** | **0** | **2** | **3** | **0** | **11** |
| **CYTB** | **8** | **5** | **9** | **7** | **0** | **29** |
| **ND1** | **3** | **4** | **3** | **7** | **0** | **17** |
| **ND2** | **4** | **2** | **5** | **5** | **0** | **16** |
| **ND3** | **4** | **2** | **5** | **1** | **0** | **12** |
| **ND4** | **1** | **2** |  | 0 | **0** | **3** |
| **ND5** | **4** | **6** | **6** | **4** | **0** | **20** |
| **ND6** | **0** | **1** |  | **3** | **0** | **5** |
| **Total** | **39** | **31** | **37** | **34** | **0** | **141** |
