# Supplementary material for: Mitochondrial Mutations in Subjects with Psychiatric Disorders
Source: PLoS One. 2015 May 26;10(5):e0127280. doi: 10.1371/journal.pone.0127280 (PMC4444211; doi:10.1371/journal.pone.0127280)
Supplement: S5 Table — There was a non-significant excess of 5+ mutations in cases compared with controls (Fishers Exact Test, one-sided p = 0.068). (DOCX) [file pone.0127280.s008.docx]

**S5 Table**. The distribution of subjects by number of non-synonymous coding mutations categorized by diagnosis. There was a non-significant excess of 5+ mutations in cases compared with controls (Fishers Exact Test, one-sided p=0.068).

|  | Number of NS mutations | 0 | 1 | 2 | 3 | 4 | 5+ |
| --- | --- | --- | --- | --- | --- | --- | --- |
|  | Subjects total | **Number of subjects with NS mutations** | | | | | |
|  |  |  | | | |  |  |
| BD | **14** | **3** | **4** | **1** | **3** | **1** | **2** |
| Controls | **20** | **4** | **5** | **3** | **4** | **4** | **0** |
| MDD | **15** | **1** | **4** | **2** | **4** | **3** | **1** |
| SZ | **14** | **3** | **2** | **2** | **3** | **2** | **2** |
|  |  |  |  |  |  |  |  |
| Cases | **43** | **7** | **10** | **5** | **10** | **6** | **5** |
| Controls | **20** | **4** | **5** | **3** | **4** | **4** | **0** |
