# Supplementary material for: Mitochondrial Mutations in Subjects with Psychiatric Disorders
Source: PLoS One. 2015 May 26;10(5):e0127280. doi: 10.1371/journal.pone.0127280 (PMC4444211; doi:10.1371/journal.pone.0127280)
Supplement: S6 Table — In gray are the variants only present in cases but not in controls. (DOCX) [file pone.0127280.s009.docx]

**S6 Table**. Homoplasmic mutations in 12S and 16S rRNA genes. In gray are the variants only present in cases but not in controls.

| **Position** | **C (20)** | **BD (14)** | **MD (15)** | **SZ (14)** | **Meth (2)** | **Total** |
| --- | --- | --- | --- | --- | --- | --- |
| 663 |  | 1 |  | 1 |  | 2 |
| 669 |  |  | 1 |  |  | 1 |
| 709 | 1 | 1 | 2 | 2 |  | 6 |
| 769 | 1 |  |  |  |  | 1 |
| 825 | 1 |  |  |  |  | 1 |
| 827 | 1 |  |  |  |  | 1 |
| 896 |  |  |  | 1 |  | 1 |
| 930 | 1 | 1 | 1 |  |  | 3 |
| 1018 | 1 |  |  |  |  | 1 |
| 1189 |  |  | 1 | 1 |  | 2 |
| 1415 |  | 1 |  |  |  | 1 |
| 1601 |  |  | 1 |  |  | 1 |
| 1700 |  |  | 1 |  |  | 1 |
| 1719 | 3 | 4 | 4 |  |  | 11 |
| 1721 |  |  |  | 1 |  | 1 |
| 1736 |  | 1 |  | 1 |  | 2 |
| 1811 | 1 |  | 2 | 2 |  | 5 |
| 1842 |  |  | 1 |  |  | 1 |
| 1861 |  | 1 |  |  |  | 1 |
| 1888 | 1 | 1 | 1 | 1 |  | 4 |
| 2092 | 1 |  | 1 |  |  | 2 |
| 2581 |  | 1 |  |  |  | 1 |
| 2702 |  |  | 1 |  |  | 1 |
| 2706 |  |  |  | 1 |  | 1 |
| 2757 | 1 |  |  |  |  | 1 |
| 2758 | 1 |  |  |  |  | 1 |
| 2885 | 1 |  |  |  |  | 1 |
| 2905 |  | 1 |  |  |  | 1 |
| 3010 | 5 | 4 | 3 | 3 | 1 | 16 |
| 3027 |  |  | 1 |  |  | 1 |
| 3096 | 1 |  |  |  |  | 1 |
| 3197 | 3 |  | 2 | 2 |  | 7 |
| 3221 |  | 1 |  |  |  | 1 |
| **Total** | **24** | **18** | **23** | **16** | **1** | **82** |
