# Supplementary material for: Mitochondrial Mutations in Subjects with Psychiatric Disorders
Source: PLoS One. 2015 May 26;10(5):e0127280. doi: 10.1371/journal.pone.0127280 (PMC4444211; doi:10.1371/journal.pone.0127280)
Supplement: S7 Table — (DOCX) [file pone.0127280.s010.docx]

**S7 Table**. Heteroplasmic variants in the DLPFC were mainly clustered in the D-Loop region.

|  |  | **Ctrl (20)** | **BD**  **(14)** | **MDD (15)** | **SCZ (14)** | **Meth**  **(2)** | **Total** |
| --- | --- | --- | --- | --- | --- | --- | --- |
| **Polypeptides** | ATP6 |  |  |  | 2 |  | 2 |
|  | ATP8 |  | 1 |  |  |  | 1 |
|  | COX1 | 1 |  |  | 1 | 1 | 3 |
|  | COX3 | 1 | 1 | 2 | 2 | 2 | 8 |
|  | CYTB |  | 1 |  | 2 | 3 | 6 |
|  | ND2 |  |  | 1 | 1 | 2 | 4 |
|  | ND3 |  | 1 |  |  |  | 1 |
|  | ND4 | 3 |  |  |  | 2 | 5 |
|  | ND5 | 1 |  |  | 1 | 3 | 5 |
|  | **Total** | 6 | 4 | 3 | 9 | 13 | 35 |
| **rRNAs** | 12SrRNA |  | 1 |  |  |  | 1 |
|  | 16SrRNA | 2 | 1 |  |  | 3 | 6 |
|  | **Total** | 2 | 2 | 0 | 0 | 3 | 7 |
| **tRNAs** | TRNL2 |  |  |  | 1 | 1 | 2 |
|  | TRNQ |  | 1 |  |  |  | 1 |
|  | TRNT |  |  |  |  | 1 | 1 |
|  | **Total** | 0 | 1 | 0 | 1 | 2 | 4 |
| **D-Loop** |  | 18 | 14 | 11 | 11 | 11 | 65 |
| **Non-coding nucleotides** |  | 2 |  | 1 |  |  | 3 |
| **Grand Total** |  | **28** | **21** | **15** | **21** | **29** | **114** |
