# Supplementary material for: Mitochondrial Mutations in Subjects with Psychiatric Disorders
Source: PLoS One. 2015 May 26;10(5):e0127280. doi: 10.1371/journal.pone.0127280 (PMC4444211; doi:10.1371/journal.pone.0127280)
Supplement: S8 Table — (DOCX) [file pone.0127280.s011.docx]

**S8 Table**. Heteroplasmic variants that were present at various levels in brain tissue but undetectable in blood.

| Position | Variant | Annotation | AA Change | MAP in brain |
| --- | --- | --- | --- | --- |
| 72 | T/C | D-loop |  | 5.3-9.6 % |
| 2487 | A/C | 16S |  | 5.5-7.9 % |
| 5755 | C/G | L-strand origin |  | 5.2-6.3 % |
| 6266 | A/G | COX1 |  | 6.4% |
| 13706 | T/G | ND5 | Leu457Arg | 5.3-5.5 % |

AA: amino acid; MAP: minor allele percentage
